# Case Report: Relief of long-standing severe motion sickness following stellate ganglion block

**DOI:** 10.3389/fnins.2026.1811989

**Published:** 2026-05-07

**Authors:** Luke D. Liu, Deborah L. Duricka

**Affiliations:** 1 Neuroversion Inc, Anchorage, AK, United States; 2 WWAMI School of Medical Education, Anchorage, AK, United States

**Keywords:** cybersickness, Mal de Débarquement, motion sickness (MS), neuromodulation, space sickness, stellate ganglion block (SGB), sympathetic nervous system (SNS), visually induced motion sickness (VIMS)

## Abstract

**Background:**

Motion sickness is a common and often debilitating condition arising from sensory conflict within the vestibular–visual–proprioceptive system, with downstream activation of autonomic and emetic pathways. Despite its prevalence in travel, maritime operations, aviation, spaceflight, and virtual environments, effective prophylaxis remains limited by adverse side effects. Experimental and clinical evidence implicates autonomic imbalance and sympathetic nervous system (SNS) hyperactivity in motion sickness susceptibility and symptom severity.

**Case presentation:**

We report the incidental resolution of long-standing, severe motion sickness in a 68-year-old man undergoing treatment with stellate ganglion block (SGB) for complex regional pain syndrome (CRPS) of the left upper extremity. The patient had experienced incapacitating motion sickness triggered by air and sea travel for approximately 28 years, substantially limiting occupational and recreational activities. He underwent a series of left-sided SGBs over several months as part of CRPS management. Following several rounds of treatment, he unexpectedly reported complete and sustained remission of motion sickness. Over 12 months of follow-up, he remained symptom-free during repeated boating activities without pharmacologic prophylaxis.

**Discussion:**

Motion sickness is associated with increased sympathetic activity, reduced parasympathetic tone during symptom provocation, and hemodynamic changes including reduced cerebral blood flow. The stellate ganglion is a major sympathetic relay to the head, neck, and thorax, and SGB is known to transiently reduce sympathetic outflow and alter cerebral perfusion. Although local anesthetic effects are short-lived, some clinical benefits of SGB appear durable, suggesting longer-term modulation of autonomic reflexes. Genetic and physiological studies further support a role for exaggerated vestibulosympathetic reflex activity in motion sickness susceptibility. Additionally, both the stellate ganglia and the central autonomic network exhibit functional lateralization, and neuroimaging data suggest a left-hemisphere contribution to motion sickness susceptibility, raising the possibility of side-specific effects.

**Conclusion:**

This case represents, to our knowledge, the first report of durable remission of chronic motion sickness following SGB. While causality cannot be established from a single observation, the finding supports further systematic investigation of SGB as a non-pharmacologic and potentially durable prophylactic treatment for motion sickness.

## 1 Introduction

Awareness of movement and spatial orientation is essential for an organism's normal functioning and quality of life. The vestibular apparatus monitors angular acceleration via the triad of semicircular canals, and linear acceleration (and gravity) via the otolith organs. Cholinergic afferent signals are sent to the vestibular nuclei in the brainstem, where additional input from the visual and proprioceptive systems are integrated to provide an accurate portrait of the organism and its temporospatial relationship to its surroundings. When contradictions occur between the visual and vestibular systems (visual-vestibular conflict), or between the components of the vestibular apparatus, motion sickness can result (Allred et al., 2025; Schmäl, 2013). Any individual with an intact vestibular apparatus is susceptible to motion sickness, although the threshold of real or illusory motion varies greatly and may be increased with training (Hu et al., 2021; Litzenberg et al., 2025; Shi et al., 2025).

Motion sickness remains a significant challenge across all forms of passive travel and simulated environments, with significant consequences for performance, safety, and operational efficiency. Individuals typically adjust within a few days of continuous exposure, but may become symptomatic again after returning to their normal environment (Ortega et al., 2019). Space motion sickness, a common occurrence in astronauts in the first 72 h in microgravity, and entry motion sickness upon returning to Earth significantly impact operational readiness and safety (Deshpande et al., 2020; Heer and Paloski, 2006). Sea sickness occurs shipboard in the majority of naval personnel, with approximately 20% requiring medication and rest in a recent study (Gupta et al., 2021). Upon returning to land, most individuals experience Mal de Débarquement—transient swaying, unsteadiness, and disequilibrium (Gordon et al., 1995). In rare cases, usually following prolonged sea travel, individuals experience these symptoms for a month or longer and are diagnosed with Mal de Débarquement Syndrome, which generally resolves within 12 months (Hain and Cherchi, 2016). Visually induced motion sickness (VIMS) in virtual environments, often referred to as “simulator sickness”, was first reported in scientific literature in 1949 as a common occurrence among air cadets training for World War II (Tyler and Bard, 1949), although earlier military documents indicate that pilots using the Link Trainer flight simulator in the 1930s experienced a similar phenomenon (Hemingway, 1943a,b) (i.e. “swing sickness”). Technological advances since then, including head-mounted displays, have resulted in the proliferation of virtual environments for entertainment and education. The term “cybersickness” was coined to describe the experience of visually induced motion sickness in immersive virtual reality, which can significantly reduce the effectiveness of this increasingly common medical educational and surgical training tool (Choi et al., 2025; El-Sourani et al., 2025; Hanke et al., 2025; Takata et al., 2021; Visan et al., 2025).

Effective prevention of motion sickness remains a critical unmet need (Koch et al., 2018; Zajonc and Roland, 2006). The first-line prophylactic for motion sickness is scopolamine, which competitively binds all types of muscarinic receptors, blocking cholinergic transmission centrally and peripherally (Qi et al., 2019; Spinks and Wasiak, 2011). However, the expected anticholinergic (specifically, antimuscarinic) adverse side effects, such as constipation and reduced cognitive performance, may also be accompanied by rebound motion sickness (withdrawal) that can persist for up to a week after discontinuing prolonged use (Lau and Vaneaton, 2014; Manno et al., 2015). Alternative pharmacological treatments for nausea include H1-antihistamines that block central cholinergic receptors, benzodiazepines, and 5-HT3 antagonists, all of which produce drowsiness inseparable from the desired effect on nausea (Singh et al., 2016). In some military settings, the sympathomimetic dextroamphetamine is added to anticholinergic or antihistamine to counteract sedation, although its usefulness is limited by the liability for abuse (Powell-Dunford and Bushby, 2017) and it has been shown to be ineffective as a treatment for motion sickness *per se* (Simmons et al., 2008). Additional psychostimulants (e.g. caffeine, Modafinil) have been investigated alone and in combination with primary anti-nausea medications with similar results (Hoyt et al., 2009; Zhang et al., 2016). Clearly there is a need for effective treatment without unacceptable side effects.

The vestibular nuclei in the brainstem integrate vestibular afferents, somatosensory and proprioceptive inputs, visceral afferents, and affective and cognitive signals, and adjust autonomic output accordingly, with modulatory input from the cerebellum (Cohen et al., 2019; McCall et al., 2017). To maintain blood distribution during postural changes, the vestibulosympathetic reflex initiates blood pressure changes within milliseconds of position changes, well ahead of the 1-second delay of the baroreflex; indeed, the expectation of motion based on cognitive and visual input can initiate peremptory hemodynamic changes via the sympathetic nervous system (Yates et al., 2014). This exquisite sensitivity makes the system vulnerable to sensory conflict during passive transport or in virtual environments. During motion sickness, nausea, pallor, cold sweat, and finally vomiting are induced by the central and autonomic nervous systems in a process that remains poorly understood (Golding, 2016). Notably, multiple studies have shown that baseline parasympathetic tone does not significantly differ between nausea-sensitive and nausea-resistant individuals, but during visually induced motion sickness, nausea-sensitive individuals exhibit increased sympathetic activity and reduced parasympathetic activity (Farmer et al., 2015, 2014; Hu et al., 1991). Vestibular stimulation results in increased cerebral vascular resistance and reduced cerebral flow velocity not accounted for by cerebral autoregulation (Serrador et al., 2009) likely mediated by vestibular-driven modulation of sympathetic outflow, which has been shown to influence both systemic and cerebral vascular tone (Hammam et al., 2011; Yates et al., 2014). Notably, increases in cardiac indices and reduction in cerebral blood flow precede and correlate with nausea induced by centrifugation and in cybersickness (Gavgani et al., 2018; Serrador et al., 2005). These observations implicate autonomic nervous system imbalance, specifically sympathetic dominance, in motion sickness.

**Figure 1:**
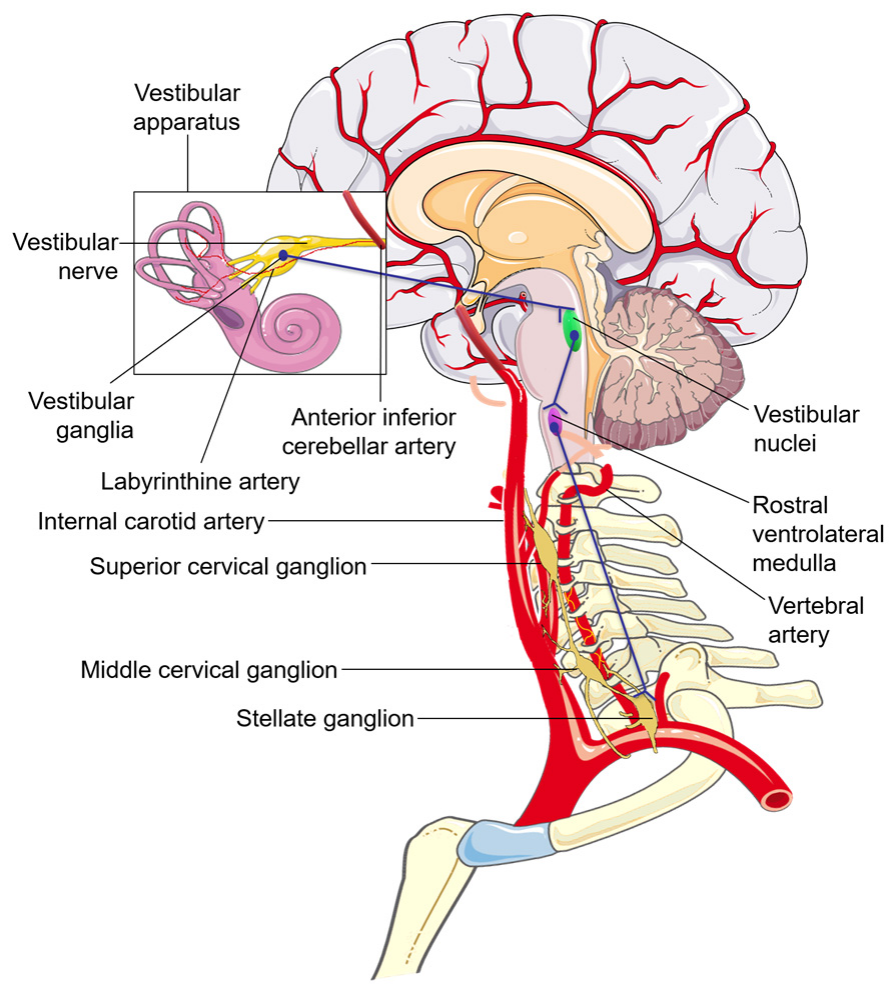
Simplified anatomical illustration of the vestibulosympathetic reflex (blue lines), cervical sympathetic chain, cerebral arterial arteries; inset box shows the vestibular apparatus with simplified innervation and vasculature. Adapted from servier medical art (https://smart.servier.com), licensed under CC BY 4.0 (https://creativecommons.org/licenses/by/4.0/).

The vestibulosympathetic reflex is mediated by projections from the vestibular nuclei to medullary autonomic centers, including the rostral ventrolateral medulla (RVLM), whose bulbospinal premotor neurons descend to sympathetic preganglionic neurons in the intermediolateral cell column of the upper thoracic spinal cord; these preganglionic neurons, in turn, innervate the cervical sympathetic chain, including the superior cervical and stellate ganglia (see Figure 1) (Holstein et al., 2014; Kulkarni et al., 2022; Moon et al., 2002). Subsequently, postganglionic adrenergic fibers innervate target organs. Efferent fibers from the stellate ganglion project to the periarterial plexus of the vertebral artery (Rusu et al., 2025), providing sympathetic regulation of vascular tone, including the vertebrobasilar system (Sun et al., 2024), which supplies the vestibular apparatus via the anterior inferior cerebellar artery and/or the labyrinthine artery (Haidara et al., 2015; Kim et al., 2022; Kim and Lee, 2009; Salgado-Lopez et al., 2020). Vascular tone is sensed by baroreceptors and relayed to the nucleus tractus solitarius (NTS), which in turn provides inhibitory or excitatory input to the RVLM (Forstenpointner et al., 2022), thereby setting the sympathetic/parasympathetic balance and baseline gain of the system controlling heartrate and vasomotor tone, i.e. the baroreflex (Yu and Chang, 2022). In a well-balanced autonomic nervous system, the sensitivity of the bidirectional baroreflex allows robust beat-to-beat regulation of heart rate, which can be modified by sympathetic block of the stellate ganglion (Keim et al., 2026).

The stellate (cervicothoracic) ganglion is formed by fusion of the inferior cervical sympathetic ganglion and the first thoracic sympathetic ganglion; this fused structure is present in roughly 80% of individuals, with significant anatomical variation in the remainder and in the shape of the stellate ganglia (Rusu et al., 2025; Samrid et al., 2024). The stellate ganglion is a major relay for sympathetic fibers supplying the ipsilateral head, neck, upper extremity, and a portion of the upper thorax including the heart and lungs. Autonomic innervation to these anatomical structures reflects a balance of sympathetic and parasympathetic inputs, the latter provided largely via cranial nerves, most notably the vagus nerve. A stellate ganglion block (SGB) involves injecting local anesthetic near the cervical sympathetic ganglion with the goal of temporarily interrupting sympathetic outflow and causing a relative increase in parasympathetic activity to these regions (Tsai et al., 2026). A successful SGB is generally indicated by ipsilateral Horner's syndrome—ptosis, miosis, and anhidrosis, although more recently pupillary ultrasonography has been used to measure changes in pupil diameter as an indicator of success (Fang et al., 2025). Clinically, SGB is used in selected conditions in which sympathetic hyperactivity is thought to contribute to symptoms, such as chronic regional pain syndrome (CRPS), Raynaud's syndrome/vasospastic circulation problems (Punj, 2019; Sahin et al., 2018), peripheral vascular disease, postherpetic neuralgia, hyperhidrosis, atypical chest pain, intractable angina (Wei et al., 2025), and refractory cardiac arrhythmia (Chouairi et al., 2024; Deng et al., 2023; Ganesh et al., 2020; Savastano et al., 2024), among others.

We treated a patient with sympathetically mediated upper extremity pain consistent with CRPS using a series of SGBs. Incidentally, the patient reported sustained remission of long-standing motion sickness that had limited his leisure activities for decades. To our knowledge, this is the first report describing durable improvement of chronic motion sickness following SGB, suggesting that SGB may warrant further study as a non-pharmacological and potentially durable treatment for motion sickness.

## 2 Case description

A 68-year-old right-handed male, 69 inches tall, with 28 BMI presented for treatment of left upper extremity pain. Specifically, he suffered from left wrist pain after traumatic injury 1 year prior to presentation. He was initially diagnosed by MRI with triangular fibrocartilage complex (TFCC) tear and a lunate cyst accompanied by loose bodies in the wrist. After approximately 8 weeks of conservative management, his injuries were treated surgically with arthroscopic synovectomy, lunate decompression, bone grafting, trapeziectomy, and ligament reconstruction and tendon interposition. Approximately 12 weeks after surgery he developed CRPS type I of the left upper extremity and was referred to our clinic for pain management.

**Figure 2:**
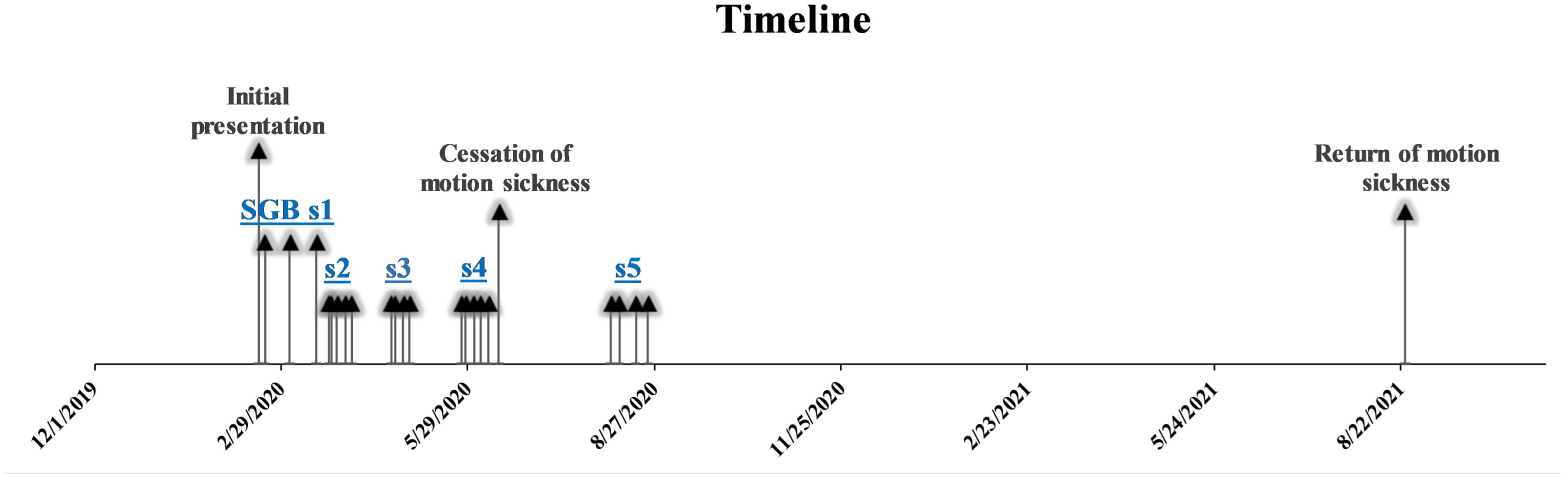
Timeline of events. SGB sX = stellate ganglion block series number X. Series 1: Left-sided SGB performed every 2 weeks. Series 2–5: Left-sided SGB performed every 1-5 days for 2 weeks.

Upon examination, the patient displayed mild to moderate hyperesthesia and allodynia over the left shoulder through the upper arm and into the hand, with increased allodynia over the metacarpophalangeal joints. We treated the patient's left upper extremity CRPS with three left-sided SGBs each separated by 2 weeks, with some improvement in range of motion but not pain (see Figure 2). We then performed left-sided SGB (for procedural details see Duricka and Liu, 2025) approximately every 1-5 days for 2 weeks (5 SGBs total) to facilitate physical therapy followed by approximately 3 weeks of observation. After approximately repeating this protocol twice, the patient reported substantial improvement in left upper extremity pain and, unexpectedly, also described absence of long-standing motion sickness during a boating excursion approximately 1 week after the fourth series of SGB treatments (see Figure 2). He reported that his motion sickness began approximately 28 years earlier during a 12-h cruise. Thereafter, he had experienced severe incapacitating symptoms with boating and air travel, with episodes sometimes persisting for up to 24 h. At that time, he received a work-up for these symptoms and was diagnosed with motion sickness after excluding relevant differential diagnoses, although records and recollection of specifics have been lost over the intervening decades, during which time motion sickness had substantially limited both his recreational activities and certain work opportunities. The patient reported remaining free of motion sickness and was able to engage in boating activities regularly over the subsequent 12 months. To address residual upper extremity pain, the patient received a final set of SGB 8 weeks following the fourth set of SGB (6 months following presentation, see Figure 2).

## 3 Discussion

Incidental observations can sometimes reveal unexpected research directions and open new avenues for investigation. During a series of left-sided SGBs performed for left upper extremity CRPS, our patient incidentally reported resolution of long-standing motion sickness. Historically, SGB has been described in the context of vertigo associated with Meniere's disease, initially as a means of predicting response to sympathectomy (Garnett Passe, 1951) and later as a therapeutic intervention in its own right (Warrick, 1969). Experimental evidence suggests adrenergic receptor activity can influence ion transport and regulate homeostasis of inner ear fluid (Kim et al., 2017), a process implicated in Meniere's pathophysiology. Indeed, clinical observations have associated sympathetic hyperactivity with onset of Meniere's attacks (Ishii et al., 2022). Although these findings do not establish a mechanism for SGB-associated clinical improvement in motion sickness, they support the broader concept that inner-ear function is closely linked to autonomic regulation.

Despite its prevalence and impact, the pathophysiology of motion sickness remains incompletely understood. An intact and functional vestibular system is generally required, but interactions with additional systems are involved in this complex process. Nausea, for example, encompasses interoceptive awareness of gastrointestinal discomfort as well as strong cognitive and emotional components (Stern, 2002; Talsma and de Winkel, 2025). Previous research has shown phasic activation of the amygdala prior to the onset of nausea (Napadow et al., 2013), while the subjective sensation of nausea is accompanied by activation of the anterior cingulate cortex, a region associated with sympathetic but not parasympathetic regulation (Beissner et al., 2013). Increased plasma epinephrine and cardiometric indices are observed during motion sickness and correlate with nausea severity (Chu et al., 2013; Farmer et al., 2015; Holmes and Griffin, 2001), further implicating SNS hyperactivity in motion sickness. Genetic data further suggest that inter-individual differences in adrenergic signaling may influence susceptibility to motion sickness. Individuals carrying a functional α_2_A-adrenergic receptor polymorphism exhibit increased motion-sickness susceptibility due to reduced presynaptic adrenergic inhibition in the brainstem, which amplifies vestibulosympathetic reflex output, leading to exaggerated sympathetic activation and enhanced autonomic/ interoceptive signaling in response to motion (Finley et al., 2004). Together with reports that indicate sympathetic predominance during motion sickness (Farmer et al., 2015, 2014; Hu et al., 1991), the effects of this polymorphism suggest that attenuating sympathetic reactivity could reduce motion sickness vulnerability.

**Figure 3:**
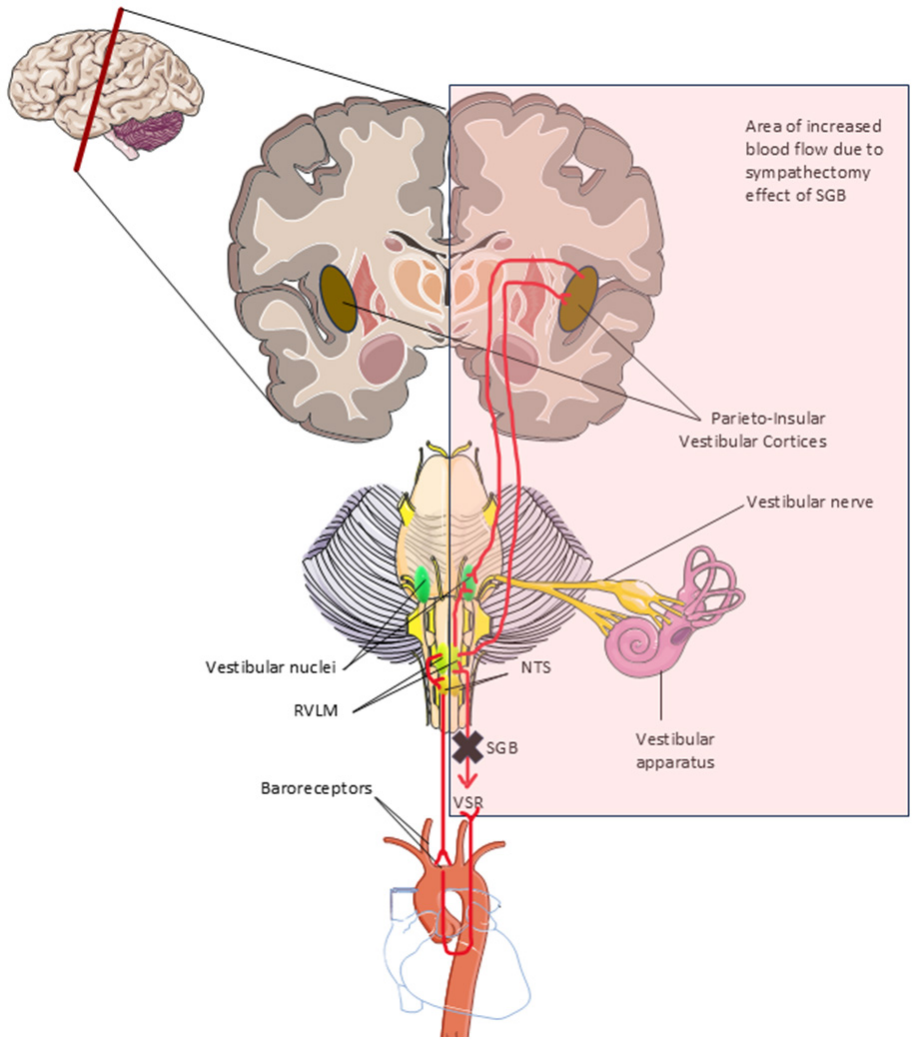
Simplified anatomical illustration of the brain regions and connections involved in the relief of seasickness following stellate ganglion block. Shaded area indicates increased ipsilateral blood flow due to sympathectomy effect of SGB. SGB, stellate ganglion block; RVLM, rostral ventrolateral medulla; NTS, nucleus tractus solitarius; VSR, vestibulosympathetic reflex. Adapted from servier medical art (https://smart.servier.com), licensed under CC BY 4.0 (https://creativecommons.org/licenses/by/4.0/).

SGB has been reported to transiently reduce circulating epinephrine and norepinephrine (Chen et al., 2017), increase cerebral blood flow (Gan et al., 2024; Kostadinov et al., 2025; Sun et al., 2024), and decrease sympathetic outflow to head and neck targets (Fang et al., 2025) yet some clinical effects seem to last well beyond the effect of local anesthesia (Duricka and Liu, 2024, 2025; Liu and Duricka, 2022; Mulvaney et al., 2014; Rae Olmsted et al., 2020). We hypothesize that SGB reduced vulnerability to motion sickness in our patient due to the convergence of the VSR and baroreflex in the RVLM, followed by interoceptive modulation of the VSR due to integration in the insular cortex (see Figure 3). The vestibulosympathetic reflex and the baroreflex form a feedforward and feedback system to regulate cardiovascular indices in response to gravitational and other changes (Chen et al., 2021; Yates et al., 2014). SGB produces hemodynamic changes sensed by baroreceptors and relayed to the NTS, which in turn modulates activity of the RVLM, adjusting the RVLM-mediated sympathetic responses to vestibular input (Yates et al., 2014). The durability of the adjustment may be due to the interoceptive system, which constantly integrates vestibular and autonomic signals as well as afferent inputs at multiple levels including the insular cortex (specifically the parieto-insular vestibular cortex, PIVC) to form a unified representation of body state and to modulate autonomic output, including the gain of the VSR (Chen et al., 2021; Evrard, 2019; Nagai et al., 2021; Owens et al., 2018). By acutely reducing sympathetic outflow, SGB may recalibrate interoceptive prediction and visceromotor set-points, durably reducing the gain of the vestibulosympathetic reflex and lowering the reactivity of the SNS, thereby decreasing susceptibility to motion sickness, as experienced by our patient. We can only speculate as to whether right-sided SGB would be similarly effective. Increased cerebral blood flow following SGB is ipsilateral (Kim et al., 2013; Park et al., 2010), and peripheral effects are also lateralized (Jiang et al., 2025)—the right side innervates the sinoatrial node with functional effects on heart rate, chronotropy and atrial arrhythmia, while the left side innervates the ventricular myocardium with effects on inotropy, contractility, and ventricular arrhythmias (Vaseghi et al., 2012). Neuroimaging studies indicate left parietal involvement in motion sickness susceptibility (Sakai et al., 2022). Indeed, the central autonomic network (CAN), a functionally connected group of brain regions (including the insular cortex) linked to the autonomic nervous system, is itself lateralized, as demonstrated experimentally in animal models, clinically in stroke victims (Fontes et al., 2024; Oppenheimer et al., 1996; Valenza et al., 2024) and in frontotemporal dementia patients (Guo et al., 2016). Hence, it is possible that left-sided SGB may effectively reduce motion sickness, while right-sided SGB may not.

Limitations of this report include the subjective reporting of seasickness severity at baseline and at resolution due to the incidental nature of the findings. The time between treatment and resolution of seasickness, along with the course of resolution (e.g. sudden vs. gradual), are unknown. Future investigation of SGB for motion sickness should include validated measures of motion sickness such as the Motion Sickness Susceptibility Questionnaire (MSSQ) and exposure to motion sickness-inducing stimuli at appropriate intervals. In addition, SGB is an invasive procedure with associated unpleasant but temporary side effects indicative of a successful block, i.e. Horner's syndrome (ptosis, miosis, and facial anhidrosis), facial warmth, and nasal congestion due to interruption of sympathetic outflow to the head and neck (Narouze, 2014). Less common but recognized complications include hoarseness from recurrent laryngeal nerve blockade, dysphagia, transient brachial plexus block, and injection-site hematoma. More serious complications are rare but may occur if the injectate unintentionally spreads to adjacent nerve structures or enters the vascular system. The latter can lead to systemic toxicity manifesting as seizures or cardiac arrhythmias. Infection, nerve injury, pneumothorax, and esophageal injury are uncommon but possible (Goel et al., 2019). The probability of these complications is strongly influenced by practitioner expertise and the use of appropriate technique. Numerous studies have shown that ultrasound or fluoroscopic guidance, careful aspiration and incremental injection, and performance by experienced clinicians substantially reduce complication rates compared with blind techniques (Goel et al., 2019; Narouze, 2014). Consequently, the overall risk profile of SGB is generally low in specialized settings but increases when the procedure is performed by less experienced operators or without image guidance. In our case, the patient received SGB for CRPS; a thorough risk-benefit discussion should occur prior to providing SGB for motion sickness *per se*.

Phenomena such as adaptation (e.g. to microgravity in space) and maladaptation (e.g. Mal de Débarquement Syndrome) clearly demonstrate that the neural systems implicated in motion sickness are amenable to short- and long-term modulation. Our observation suggests that targeted suppression of SNS activity with SGB may be associated with sustained resolution and prevention of motion sickness without the undesirable anticholinergic side effects associated with current pharmacological agents. Given the prevalence and functional impact of motion sickness, this incidental finding warrants further research and systematic investigation of SGB as a potential non-pharmacologic intervention.

## Data Availability

The original contributions presented in the study are included in the article/supplementary material, further inquiries can be directed to the corresponding author.
